# Super-enhancer profiling reveals ThPOK/ZBTB7B, a CD4+ cell lineage commitment factor, as a master regulator that restricts breast cancer cells to a luminal non-migratory phenotype

**DOI:** 10.21203/rs.3.rs-6240646/v1

**Published:** 2025-03-31

**Authors:** Denise Paula Muñoz, Camila D Arcuschin, Kamin Kahrizi, Rosalyn W Sayaman, Carolina DiBenedetto, Pedro J Salaberry, Yizhuo Shen, Ons Zakroui, Cecilia Schwarzer, Alessandro Scapozza, Paola Betancur, Julie D Saba, Jean-Philippe Coppé, Mary-Helen Barcellos-Hoff, Dietmar Kappes, Laura van ‘t Veer, Ignacio E Schor

## Abstract

Despite efforts to understand breast cancer biology, metastatic disease remains a clinical challenge. Identifying suppressors of breast cancer progression and mechanisms of transition to more invasive phenotypes could provide game changing therapeutic opportunities. Transcriptional deregulation is central to all malignancies, highlighted by the extensive reprogramming of regulatory elements that underlie oncogenic programs. Among these, super-enhancers (SEs) stand out due to their enrichment in genes controlling cancer hallmarks. To reveal novel breast cancer dependencies, we integrated the analysis of the SE landscape with master regulator activity inference for a series of breast cancer cell lines. As a result, we identified T-helper-inducing Poxviruses and Zinc-finger (POZ)/Krüppel-like factor (ThPOK, ZBTB7B), a CD4+ cell lineage commitment factor, as a breast cancer master regulator that is recurrently associated with a SE. ThPOK expression is highest in luminal breast cancer but is significantly reduced in the basal subtype. Manipulation of ThPOK levels in cell lines shows that its repressive function restricts breast cancer cells to an epithelial phenotype by suppressing the expression of genes involved in the epithelial-mesenchymal transition (EMT), WNT/b-catenin target genes, and the pro-metastatic TGFb pathway. Our study reveals ThPOK as a master transcription factor that restricts the acquisition of metastatic features in breast cancer cells.

### Introduction

In recent years, breast cancer mortality has declined due to broader screening, early detection, better classification, and the discovery of new targeted therapies. However, metastatic breast cancer remains incurable, and many patients lack effective treatments (1, 2).

Breast cancer is classified based on the expression of different receptors: estrogen and/or progesterone receptors (ER/PR+), amplification or overexpression of epidermal growth factor 2 (HER2+), or the absence of these three markers in the case of triple negative breast cancer (TNBC). However, this classification is complemented by the molecular intrinsic subtyping based on transcriptional profiles that form the PAM50 signature and divides them into luminal A (Lum A), luminal B (Lum B), Her2-enriched (HER2+), basal-like and normal-like subtype (3, 4).

Both genetic and epigenetic factors contribute to the heterogeneity of breast cancer, with aberrant epigenetic changes driving cancer progression. Super-enhancers (SEs), highly active regions of clustered enhancers, are emerging as key regulatory elements in tumorigenesis, aiding in diagnosis and classification, helping understand subclonal evolution, and predicting drug responses (5, 6). These regions are characterized by unusually high signals of histone modifications such as H3K27ac and H3K4me1, and chromatin regulatory proteins such as BRD4, the mediator complex, and cell-type specific transcription factors (TF) (7, 8). SEs control the expression of genes essential for normal cells' identity, and in cancer cells regulate genes involved in survival, oncogenesis and drug resistance (7-12). This suggests that identifying SEs and their associated genes can reveal previously unappreciated cell dependencies and vulnerabilities, as well as inform about cancer biology, clinical diagnosis and therapeutic guidance (13). In breast cancer cells (BCCs), SEs regulate genes involved in immune evasion (14) and therapeutic resistance (15, 16), and their perturbation by bromodomain and extraterminal (BET) protein inhibition restrains proliferation and promotes apoptosis (12, 17).

ThPOK (**T**-**h**elper-inducing Poxviruses and Zinc-finger (**PO**Z)/**K**rüppel-like factor), encoded by the *ZBTB7B* gene, is a transcription factor that belongs to the POK family of transcriptional repressors containing BTB/POZ domains. It was originally known for regulating extracellular matrix (ECM) genes in fibroblasts (18, 19) and CD4+ T lymphocytes lineage commitment (20), but its role in other contexts is less well known. While other members of the POK family have been implicated in carcinogenesis (21), there is no such oncogenic function described for ThPOK under non-manipulated circumstances. However, transgenic mice constitutively expressing ThPOK in T cells (T-cell specific *ThPOK* transgene, *ThPOK*$^{const}$ mice) develop thymic lymphomas resembling human T-cell acute lymphoblastic leukemia (T-ALL) (22). Only recently, ThPOK has been implicated in regulating lipid synthesis and lipid droplet secretion by the lactating murine mammary gland (23).

Here, we reveal a novel role for ThPOK as a master regulator in BCCs. The ThPOK/*ZBTB7B* gene is associated with a SE in cells derived from different breast tumor subtypes, a feature that distinguishes key transcriptional regulators in cancer. ThPOK expression levels are higher in breast tumors compared to normal adjacent tissues, with the highest levels observed in cells/tumors of the luminal subtype.

Conversely, a major repressed status is observed in TNBC cell lines or tumors, likely driven by specific CpG methylated sites. Manipulation of ThPOK levels revealed a role for ThPOK in limiting BCCs plasticity to an epithelial non-migratory phenotype by restricting the expression of ECM genes, epithelial-mesenchymal transition (EMT) factors, and WNT and TGF$\beta$ pathways.

### RESULTS

#### Ubiquitous super-enhancers associate with key regulators of gene expression in breast cancer cell lines

Since SEs control oncogenes and regulators of cellular identity (5, 7, 24), we reasoned that characterizing SE profiles in both normal and cancer cells can reveal cancer-specific dependencies. To map the regulatory landscape of breast cancer, we used chromatin immunoprecipitation followed by sequencing (ChIP-seq) to determine the genome-wide distribution of H3K27ac in BCC lines derived from primary tumors. We combined our data with available H3K27ac ChIP-seq datasets from different BCC lines. After quality control of 80 input/H3K27ac ChIP-seq datasets, we analyzed 37 samples from 17 breast cancer cell lines (**Table S1**), identifying individual H3K27ac peaks and SE regions with MACS and ROSE2 algorithms respectively (11, 25). The consolidated set includes 5917 SEs (**Fig. 1a**) proximal (+/-10Kbp) to 9911 genes. Our analysis confirmed that the 200 most variable SE regions (**Table S2)** cluster the cell lines according to their subtypes with few exceptions (**Figs. S1a-b**). Functional analysis of the complete SE set using the Genomic Regions Enrichment of Annotations Tool (GREAT) (26) showed SE-associated genes enriched in terms related to cell differentiation, mRNA metabolism, cell signaling and cell architecture (**Fig. S1c** and **Table S3**). In summary, using this strategy, we obtained a consolidated SE set that reflects known grouping of the cell lines and at the same time is associated with genes with functions relevant to carcinogenesis.

While many SEs within this set are only found in one cell line (cell line-specific SE, 3119), we identified a subset (126) of ubiquitous SEs that are common across at least two-thirds of the cell lines (**Fig. 1b**). GREAT analysis revealed that genes associated with ubiquitous SEs, unlike cell line-specific SEs, are significantly enriched in terms related to gene expression regulation, chromatin remodeling, differentiation, metabolism and apoptosis (**Fig. 1c** and **Table S4**). In addition, ubiquitous SEs are also specifically linked to BCC core regulatory circuitry genes (**Fig. 1d**, dark grey bars) defined in Saint-André *et al* (24). In conclusion, in contrast to cell line-specific SEs, we found that ubiquitous SEs are associated with key regulatory elements of breast cancer cell biology. Therefore, we focused on this subset for further analysis.

#### ThPOK/*ZBTB7B* as a breast cancer master regulator repressed in TNBC

To prioritize key genes and cellular states, we used the ubiquitous SE set and integrated it with RNA-seq data from BCCs (27) (**Table S5**) to identify crucial transcriptional regulators with subtype-specific activity. We employed VIPER (28) to analyze a breast cancer gene regulatory network built from The Cancer Genome Atlas (TCGA) transcriptomic data (ARACNe-BRCA) (29). This revealed the activity patterns of 4438 breast cancer master regulators (MRs) by monitoring their downstream transcriptional targets expression (i.e. regulons) (**Fig. 1e**). By comparing each subtype (Lum, HER2+, TNBC and normal (Nor)) to the rest, we calculated a normalized enrichment score (NES)

for each MR in each subtype (**Table S6**). We focused on MRs with differential activity of absolute NES > 2 and genes associated with ubiquitous SEs, identifying 41 MR candidates (**Fig. 1e**, right panel). Many showed differential activity that could be due in part to variations in their own expression (**Fig. 1e**, compare activity (NES) vs RNA level). Notably, ThPOK (*ZBTB7B*), a $CD4^{+}$ lineage commitment transcription factor, was significantly under-active in TNBC and over-active in luminal cell lines. This gene is linked to a large ubiquitous SE (**Fig. 1f**, red bar), though the H3K27ac signal near the transcriptional start site is lower in TNBC lines (**Fig. 1f, Fig. S2a-b**). To confirm SE regulation of ThPOK, we inhibited BRD4, an essential SE component (7, 17, 30-32), using JQ1 or iBET-151. Acute BRD4 inhibition (6h) reduced *ThPOK* expression (**Fig. S2c, left**), without inhibiting the housekeeping gene *ACTB* (**Fig. S2c, right**) or ectopic *ThPOK* expression through a stable integrated lentiviral vector (**Fig. S2d**). Moreover, ThPOK fits the profile of a master regulator: it regulates gene expression, recruits chromatin repressors (33, 34) and influences cell lineage phenotypes (20, 35). Given its oncogenic potential within the POK protein family, we investigated the specific role of ThPOK in breast cancer.

### ThPOK is upregulated in luminal breast cancer cells and tumors

Quantitative RT-PCR (qRT-PCR) analysis of *ThPOK* expression across 21 breast cancer cell lines revealed subtype-specific differences in mRNA levels: highest in luminal cell lines, lowest in TNBC lines, and intermediate yet more heterogeneous in HER2+ cell lines (**Fig. 2a**). These findings were consistent with RNA-seq data from various cell lines (**Fig. 2b**) and suggested that despite the ubiquitous presence of the SE linked to *ZBTB7B/ThPOK* (**Fig. 1f**), subtype-specific regulatory mechanisms modulate its expression, resulting in distinct quantitative mRNA profiles. At the protein level, immunofluorescence confirmed a similar subtype-associated pattern of ThPOK protein expression (**Fig. 2c**). Using the UALCAN portal (36, 37), analysis of RNA-seq profiles from patient samples in TCGA revealed higher *ThPOK* expression in breast tumors compared to adjacent normal tissue (**Fig. 2d**). Subtype-specific comparisons indicated that luminal hormone receptor + (HR+) and HER2+ tumors exhibited higher *ThPOK* mRNA levels than TNBC tumors (**Fig. 2e**). Analysis of single cell RNA-seq data from ER+ tumors (38) further demonstrated high *ThPOK* expression specifically in cancer epithelial cells (**Fig. 2f**). Detailed analysis of TCGA data, incorporating PAM50 classification (4) and hormone receptor status determined by immunohistochemistry (**Fig. S3a-b**), showed the highest *ThPOK* mRNA levels in PAM50 LumA and HER2+ subtypes (**Fig. S3a**). In contrast, more aggressive PAM50 LumB tumors had lower *ThPOK* levels (**Fig. S3a**). Among receptor subtypes, $ER^{+}$ tumors showed the highest *ThPOK* levels, with HER2+ tumors displaying the highest expression in the $ER^{-}$ group (**Fig. S3b**). Epigenetic analysis revealed increased CpG methylation at several sites within the *ZBTB7B* 5' UTR in TNBC tumors, suggesting transcriptional repression mediated by CpG methylation (**Figs. S3c-e)**. Supporting the significance of ThPOK's role in breast cancer, data from the Human Protein Atlas highlighted that breast cancer exhibited the highest ThPOK protein levels and ranked second for mRNA expression across cancer types (**Figs. S4a-b**). Clinical relevance of ThPOK activity was further supported from the analysis of an independent dataset, the METABRIC cohort (39). Consistent to what we observed in TCGA expression data (**Fig. S3b**), this analysis indicated that ThPOK's activity is indeed elevated in ER+ tumors (**Fig. S5a**). Notably, and specific to ER+ tumors, low ThPOK activity was associated with worse prognosis (higher Nottingham Prognostic Index) (**Fig. S5b**), higher probability of positive lymph nodes (**Fig. S5c**) and lower relapse-free survival (**Fig. S5d**). Additionally, we observed that deletions of the *ZBTB7B* gene correlated with shorter time to metastasis (**Fig. S5e**, Metastatic Breast Cancer Project data).

**ThPOK silencing de-represses a pro-migratory gene expression program**

Although ThPOK is mainly known as a transcriptional repressor, depending on the context it can also act as an activator (33, 40-42). To understand ThPOK's specific action and targets in BCC, we used shRNA-mediated knockdown (kd) to reduce its mRNA levels (**Fig. 3a, S6a**) in luminal (T47D, MCF7 and ZR75.1) and HER2+ (BT474) cell lines with high-to-intermediate *ThPOK* mRNA levels (**Fig. 2a**). We initially assessed the expression levels of known ThPOK target genes in fibroblasts and T cells, which may have a role in breast cancer. Reduced ThPOK levels led to increased ECM gene expression (*FN1*, *COL1A1*, *COL1A2*) (**Fig. 3b**), and caused EMT-like morphological changes in most cell lines (**Fig. 3c, S6b**). Since ThPOK has features of a master regulator, we assessed the impact of its silencing on other gene sets representing different cancer hallmarks. ThPOK-kd led to an increase in the expression of EMT factors (*VIM*, *SNAI2*, *ZEB1*, *ZEB2*), basal markers and WNT/β-catenin target genes implicated in breast cancer cell proliferation (**Fig. 3d-f**) (43, 44). The protein membrane and stemness marker CD44 was also upregulated as shown by qRT-PCR and flow cytometry analysis (**Fig. 3f-g, S6c-d**). Consistent with this, using TCGA breast tumor's genomic data, we found that low levels of ThPOK correlate with a higher RNA-based stemness score (45) (**Fig. S6e**). We didn't observe a consistent change in expression of epithelial markers (Fig. **3h**) but the *ESR1* gene, coding for the ER, was upregulated both in luminal and HER2+ cell lines (**Fig. 3i**). In agreement with the observed alteration of their transcriptional programs, luminal cells with reduced levels of ThPOK exhibited an enhanced migratory phenotype (**Fig. 3j-k** and **S6f**).

**ThPOK expression induces a "luminal-like" gene signature in HER2+ and TNBC cell lines**

Based on our previous results, we hypothesized that overexpressing ThPOK in HER2+ and TNBC cells, with lower endogenous ThPOK levels, would cause opposite changes in gene expression patterns and cellular phenotypes. We transduced HCC1954 (HER2+), and HCC1143, BT549 and MDA-MB-231 (TNBCs) with a lentivirus encoding ThPOK variant #5, the most abundant variant expressed in BCCs (**Fig. S7a-b)**. ThPOK overexpression ($ThPOK^{OE}$) was confirmed by qRT-PCR and Western Blot (**Fig. 4a & Fig. S8a**). As predicted, ThPOK overexpression decreased ECM genes expression and increased epithelial markers including *CDH1*, while decreasing the expression of genes associated with migration and invasiveness, such as EMT markers (*VIM*, *SNAI1*, *SNAI2*, *ZEB1*, *ZEB2* and *TWIST*), WNT/β-catenin target genes, the stemness marker *CD44* and *ESR1* (**Figs. 4b-g**). These changes are accompanied by a mesenchymal to epithelial transition-like phenotype and reduced migratory features (**Figs. 4h-i & S8b**).

Taken together, these data indicate that ThPOK functions as a transcriptional repressor of genes that are critical for breast cancer phenotypes associated with invasiveness and aggressiveness.

**ThPOK inhibits the TGFβ pathway but independently regulates ECM genes**

Since the expression of *FN1* and *COL1A1* genes is positively regulated by TGFβ signaling (42), we hypothesized that the repressive action of ThPOK over these ECM genes could be through downregulation of the TGFβ pathway, a central regulator of ECM remodeling and fibrosis. Indeed, when we analyzed the levels of TGFβ/SMAD target genes in breast cancer cell lines from the Cancer Cell Line Encyclopedia (CCLE) we saw an inverse correlation between the expression of *ThPOK* and of TGFβ target genes (**Fig. 5a**). By measuring TGFβ (*TGFB1*) mRNA levels in ThPOK-kd (**Fig. 5b**) and $ThPOK^{OE}$ cells (**Fig. 5c**) by qRT-PCR, we confirmed that ThPOK is a repressor of TGFβ mRNA expression in BCCs. TGFβ is produced as a latent complex and is activated extracellularly by many factors (46). To test whether ThPOK-kd cells can activate the produced TGFβ, control cells were exposed to conditioned media collected from ThPOK-kd cultures in the presence or absence of a TGFβ receptor I (TβRI) inhibitor (LY2157299), and expression of *FN1* and *COL1A1* genes was assessed. Conditioned media from ThPOK-kd cells significantly increased ECM genes' mRNA levels in luminal/HER2+ cells, and this effect was mostly mitigated by TβRI inhibition (**Fig. S9a**), indicating that ThPOK-kd cells produce and activate TFGβ, which may have autocrine as well as paracrine effects on ECM genes' expressions.

We hypothesized that if ThPOK represses ECM genes by repressing the expression of TGFβ, ThPOK action should be bypassed by exogenous TGFβ treatment. However, while treatment of luminal cancer cell lines with recombinant TGFβ (rTGFβ) increased *FN1* and *COL1A1* expression, the magnitude of the effect was smaller, and a longer treatment was needed in cells with high endogenous ThPOK levels (T47D), compared to cells with lower levels (MCF7) (**Figs. 5d** and **S9b**). Similarly, rTGFβ treatment only partially counteracted the effects of ectopic ThPOK overexpression in TNBC for the assessed ECM genes (**Figs. 5e** and **S9c**). These results suggest that in both scenarios, the repression imposed by high levels of endogenous and/or ectopic ThPOK in the expression of ECM genes it can only be partially counteracted by the inducing effects of TGFβ signaling, suggesting a direct regulatory role of ThPOK on these genes, besides its action on *TGB1* expression. To further confirm this, we inhibited TβRI activity in ThPOK-kd luminal and HER2+ cells, so that any autocrine signaling would be blunted, and evaluated *FN1* and *COL1A1* expression. Although TβRI inhibition slightly reduced the expression of TGFβ target genes, it did not fully revert their levels to those of the original scramble control cells (**Fig. 5f**, compare Veh vs kd+LY). This implies that while TGFβ produced by ThPOK-kd cells may autocrinally contribute to some extent to the increase in ECM gene expression when ThPOK levels are reduced, the primary driver of their dysregulation is the absence of ThPOK's repressive functions.

In summary, our studies highlight ThPOK as a key transcriptional regulator that limits breast cancer cells plasticity by repressing TGFβ, ECM genes and mesenchymal factors, thereby promoting an epithelial, non-migratory phenotype (**Fig. 5g**).

### DISCUSSION

Epigenetic dysregulation is a hallmark of cancer, and the identification of SEs has aided in the discovery of unexpected dependencies and novel drug targets (8, 32). In this study we employed a multi-omic approach that integrates SE profiling and transcriptomic analysis of 17 normal mammary and breast cancer cell lines with a breast cancer-specific gene regulatory network, revealing ThPOK/*ZBTB7B* as a subtype-specific master regulator in breast cancer. A key step in our analysis was the prioritization of a small subset of SEs, ubiquitous SE, which consistently showed SE features in the different BCC lines assessed and were enriched in the proximity of key cell identity genes. We believe that selecting for commonalities between cell lines helped us to avoid epigenetic abnormalities that may arise in specific cell lines and do not necessarily represent properties of that tumor type.

At the same time, even though the *ZBTB7B* gene was associated with an ubiquitous SE, it showed clear subtype-specific expression. This may indicate that SEs impart complex regulatory architecture rather than solely controlling gene expression, potentially allowing chromatin accessibility at the ThPOK locus permitting fine-tuning to adapt to the changing demands of the lactating mammary gland. Our discovery of ThPOK as a master regulator is further supported by its role as a methylation modulator gene in breast invasive carcinoma (47). An integrated analysis of DNA-methylation and RNA-seq data from ~500 breast invasive carcinoma samples features ThPOK as a hub node within a gene module containing several genes regulated by WNT/β-catenin and TGFβ (47).

ThPOK's physical interaction with histone deacetylases (HDACs), and promoters of non-transcribed genes suggests that ThPOK primarily functions as a transcriptional repressor (33, 40). Consistent with these findings, we find that ThPOK represses genes involved in ECM composition, as well as transcription factors implicated in the EMT and WNT/β-catenin signaling pathways in breast cancer cells. These effects coincided with cellular morphological and behavioral changes indicative of enhanced migratory capacity. In addition, we identified a common set of genes regulated by ThPOK and polycomb repressor complex 2 (PRC2) in transformed mammary epithelial cells (48). Dysfunction of PRC2 and ThPOK deficiency, both lead to a quasi-mesenchymal state, characterized by de-repression of many EMT and basal genes and retention of some epithelial features. We are currently investigating whether ThPOK associates with polycomb repressor family of proteins to exert its repressive functions.

The ECM influences cancer cell behavior through chemical and mechanical signals, with changes in collagen composition linked to tumor invasion and aggressiveness (49). Recent studies have shown that collagen deposition and organization regulate dormancy in disseminated breast cancer cells and immune exclusion in TNBC (50, 51). Given ThPOK's repression of ECM genes, the regulation of ThPOK expression could profoundly impact the ECM and consequently alter cancer cell behavior. This is also consistent with the fact that ThPOK regulates *TGFB1* mRNA and TGFβ signaling pathways which play a critical role in tumor formation, progression and metastasis (44). Notably, *FN1* and *COL1A1*, all regulated by TGFβ (52, 53), exhibit coordinated expression in breast cancer lymph node metastases (53). These proteins form networks that promote a spindle-like phenotype, significantly enhancing tumor cell migration. While ThPOK-mediated repression of ECM genes could occur indirectly through the autocrine effects of TGFβ, our findings and previous studies suggest a direct role of ThPOK in the transcriptional inhibition of these genes, indicating that it is at least partially independent from TGFβ regulation (18, 19, 54).

A complex interplay among GATA3, RUNX and ThPOK determines T cell fate, with GATA3 as an upstream positive regulator of ThPOK in the thymus (55) and ThPOK influencing GATA3 expression in breast cancer cells. GATA3, a critical regulator of luminal differentiation during mammary gland development, also acts as a key transcription factor in maintaining an epithelial phenotype in luminal breast cancers (56-58). Interestingly, ThPOK levels are similarly elevated in this subtype, supporting the idea of a functional interaction between GATA3 and ThPOK where they may collaborate to maintain luminal identity in both normal and cancerous cells (56, 57). Moreover, GATA3 participates in a positive feedback loop with ER$\alpha$ in breast cancer (57), and ThPOK interacts with ER$\alpha$ in the nuclei of breast cancer cells (59). Exploring the interplay among ThPOK, GATA3 and ER$\alpha$ could provide insights into breast cancer progression, and luminal differentiation and maintenance.

A subset of ductal carcinoma in situ (DCIS) cases progress to invasive ductal carcinoma (IDC), posing clinical challenges in identifying patients requiring a more aggressive treatment. Investigating ThPOK repression as a mechanism facilitating the acquisition of more aggressive phenotypes could shed light on the transition to invasive disease.

In conclusion, our multi-omic approach uncovered ThPOK, known as a $CD4^+$ lineage commitment factor, as a breast cancer master regulator, with manipulation of ThPOK levels revealing a network of target genes enriched in cellular plasticity programs. Understanding how cancer cells regulate repressive transcription factors like ThPOK, which absence can become a tumor-promoting factor, is critical for mitigating carcinogenesis and tumor progression.

### MATERIALS AND METHODS

#### Cell lines, culture conditions, constructs and lentivirus production and transduction

HCC1954, BT20, BT483, HCC1569, and BT474 breast cancer cell lines were bought from ATCC. MDA-MB-361 and MDA-MB-453 were bought from UC Berkeley Cell Culture laboratory. ZR75.1, MDA-MB-436, and ZR75.B were kindly given by Dr. Desprez. MCF10A, MCF7, T47D, MDA-MB-231, and SKBR3 were kindly given by Dr. Kohwi-Shigematsu. HMLER cells were kindly provided by Dr. Weinberg. All cell lines tested negative for Mycoplasm, were grown at 5%$CO_2$ with all media was supplemented with 10% FBS and 1X Penicillin/Streptomycin. BT20, BT474, HS578T, MCF7, MDA-MB-231, MDA-MB-453, MDA-MB-361, MDA-MB-436, and SKBR3 were grown in DMEM; BT483, BT549, HCC1143, HCC38, HCC70, HCC1569, HCC1954 and ZR75.1, MDA-MB-436, ZR75.B, and T47D in RPMI. MCF10A and HMLER cells were grown as described (60, 61).

#### ThPOK construct

*ThPOK* mRNA variant #5 (NCBI Reference Sequence: NM_001256455.2) was amplified by PCR from cDNA and cloned into pENTR1A-DS vector (Invitrogen) using SalI and NotI sites, then sequenced for accuracy. The pENTR1A-ThPOK vector was recombined into pLenti-CMV-DEST-hygro (W117-1) (provided by Dr. Campeau (62)) using Clonase II (Thermofisher, cat# 11791).

#### Lentivirus production and transduction shRNA-expressing lentiviral vectors for ThPOK knockdown were purchased from Open Biosystems/Dharmacon (RHS4533-EG51043). Lentiviruses containing shRNA or

ThPOK constructs were produced as described in Rodier *et al* (63). Transductions were done overnight with 4µg/ml of Polybrene™. 48h later cells were selected with 2µg/ml puromycin or 400µg/ml hygromycin.

**Chromatin immunoprecipitation and next generation sequencing (NGS)**
Chromatin preparation and immunoprecipitation were performed as described in (64) using a H3K27ac antibody (Abcam cat# Ab4729). ChIP-seq libraries were prepared using Illumina protocols, quantified with Kapa Biosystems kits, pooled and sequenced on a HiSeq4000 as 50-base single reads.

**NGS data analysis**
RNA-seq data were quality controlled with FastQC. Reads were aligned and transcript counts were obtained using kallisto with 100 bootstraps (65). Master regulator activity inference from RNA-seq data was performed with VIPER (28). ChIP-Seq data were quality controlled with FastQC, mapped using Bowtie 2.0, and H3K27ac peaks and SE were obtained using MACS2 and ROSE2 algorithms (7, 8, 25). For more detail refer to supplementary methods.

**Analysis of single cell RNA-seq data from tumors**
Processed scRNA-seq data of 11 IHC-inferred ER+ breast cancer tumors from (66) was downloaded from the Gene Expression Omnibus (GEO) under accession number GSE176078 and was analyzed in R with the Seurat suite (67) using standard pipelines. Gene expression data of each tumor was normalized using scTransform and consequently integrated with the canonical correlation analysis (CCA) method using default parameters. Cell type identification was taken from the original paper. To visualize the single-cell data in a reduced-dimensional space, we performed Principal Component Analysis (PCA) and Uniform Manifold Approximation and Projection (UMAP) with 20 dimensions and k.param = 20 for FindNeighbors and n.neighbors = 20 for RunUMAP. Plots were generated with the functions DimPlot and FeaturePlot. *ZBTB7B*/ThPOK levels were expressed in normalized counts by scTransform.

**Immunofluorescence**
ThPOK immunofluorescence was performed as previously described (68), using an antibody from Novus Biologicals cat# NBP1-88077. Secondary antibody was anti-rabbit IgG ReadyProbes™ Alexa Fluor 594. Nuclei were counterstained with DAPI. Images were taken using the same exposure time using a Keyence fluorescence microscope BZ-X800 at 400X magnification.

**Western-blot**
Cells were lysed and proteins separated by SDS-PAGE. Primary antibodies: ThPOK (Novus Biologicals cat# NBP1-88077); GAPDH (Cell Signaling cat# 97166); β-Actin (Abcam cat# ab6276). Secondary antibodies: anti-mouse and anti-rabbit HRP-conjugated (BioRad cat# 1705047EDU and 1662408EDU).

**RNA extraction, cDNA synthesis and qRT-PCR**
RNA was purified with TRIzol followed by DNAse RNase-free digestion (Qiagen). cDNA synthesis was performed using the High-Capacity cDNA Reverse Transcription kit (ABI). qRT-PCR primers sequences will be provided upon request. qRT-PCR was performed using SYBR-Green Power master mix (Thermofisher), analyzed with the QuantStudio5

software, and relative mRNA levels were calculated using the $2^{(-\Delta\Delta Ct)}$ method using *GAPDH* and *HPRT1* as controls. We used a *t*-test for two-samples with equal variance (with a two-tailed distribution) to test the significance of gene expression differences.

**Flow cytometry**
For CD44 staining, cells were collected, washed in FACS buffer (2% FBS in PBS), and kept at 4ºC for the rest of the protocol. Cells were stained for 1 hour with an anti-CD44-PE antibody (Cell Signaling, cat# 88151) at a 1:300 dilution in FACS buffer. After washing, cells were stained for 20 minutes with LIVE/DEAD Fixable Violet Dead (Life Technologies) at a 1:7500 dilution in PBS. After washing, CD44+ events were quantified using a Northern Light cytometer (Cytek). Data were analyzed on SpectroFlo software (Cytek).

**Scratch and Boyden chamber assays**
For the scratch assay, once cells reached confluency three parallel scratches were made per well. After 48h, cells were fixed with 4% (V/V) paraformaldehyde, stained with 0.1% Crystal Violet for 20min, rinsed and air-dried. The scratches were imaged using a Nikon Eclipse TS100 light microscope under 4x objective. Multiple and overlapping images were taken along each scratch. Image J was used to measure the area unoccupied by cells, which was reported under the measurement parameter "Area". We created Macro Plug-in with a code to analyze the images in batches. Data from individual images were grouped together per scratch. Images with highly irregular shapes and of the head and the tail region of the scratches were excluded. The assay results of each scratch were calculated by taking the mean of those grouped images, and then plotting based on different treatments. Data were obtained from at least 3 biological replicates. Boyden chamber assay was performed as described in (68) with no Matrigel and 10% FBS-media as chemoattractant. Cells on the outside bottom of the wells were fixed with 4% paraformaldehyde, stained with 0.1% crystal violet, extracted with 10% acetic acid and quantified at 590nm. Experiments were done three times in triplicates.

**Treatment with TGFβ and inhibition of TGBRI**
Cells were treated with 10ng/ml of rTGFβ (Life Technologies, cat#: PHG9204) for the indicated times. To assess the activity of TGFβ produced by ThPOK-kd cells, the cells were grown in RPMI/DMEM with 10% of Serum Replacement Medium (KO SRM Gibco cat# 10828-028) for 72h before the supernatant was collected, centrifuged and filtered to constitute the conditioned media (CM). shScr cells were grown in the presence of conditioned media with or without the TGBRI inhibitor LY2157299, at a 2nM concentration for 24h. To assess TGFβ's autocrine function, cells were grown in RPMI/DMEM with 10% SRM for 24h with or without the TGBRI inhibitor LY2157299 at a 2nM concentration.

**GSEA and stemness score analyses**
CCLE and TCGA datasets were used to calculate Spearman correlations between *ZBTB7B* and the rest of the genes, and the correlation-ranked genes were analyzed for enrichment of TGFβ target genes (69) using GSEA (70) within the clusterProfiler R package (71). TCGA BRCA data on *ZBTB7B* RNA expression and RNA-based stemness score was downloaded from Xena browser (72). Samples were divided in two groups based on the median value of *ZBTB7B* RNA expression. t-test with Welch's correction was performed.

**Statements and Declarations**

### Funding

This work was supported in part by National Institutes of Health (NIH/NCI) [5K22CA163969-02], Pink Pumpkin Patch, Swim Across America, Breast Research Development Program (UCSF), and RAP (UCSF Resource Allocation Program) grants to DPM, grants from the Agencia Nacional de Promoción de Ciencia y Tecnología of Argentina and the Universidad de Buenos Aires to IES, and by NIH/NCI Cancer Metabolism Training Program Postdoctoral Fellowship [T32CA221709] and Mentored Research Scientist Development Award to Promote Diversity [K01CA279498] to RWS.

### Competing Interests

The authors have no relevant financial or non-financial interests to disclose.

**Author Contributions**

DPM and IES designed research; KK, CD, YS, OZ, CS, AS, and DPM performed research; CA, KK, RWS, YS, PS, PB, M-H B-H, J-PC, IES and DPM contributed new reagents/analytic tools; CA, KK, RWS, YS, OZ, PS, JDS, J-PC, LvV, DK, IES and DPM analyzed data. The first draft of the manuscript was written by DPM and IES and all authors commented on previous versions of the manuscript. All authors read and approved the final manuscript.

**Data availability**

Data publicly available analyzed in this study is listed with their references in Supplementary Tables 1 & 5. Datasets generated in the current study have been deposited in the GEO repository, https://www.ncbi.nlm.nih.gov/geo/query/acc.cgi?acc=GSE207245.

**Acknowledgments**

We thank Diane Heditsian, our breast cancer patient advocate for her priceless support and discussions of patients' needs to ensure we fairly represent breast cancer patients in our research, and Martín García Solá for his support and advice.

### Figure legends

**Figure 1. Combining ubiquitous SE identification with subtype-specific master regulator activity analysis helps identification of candidate key regulators of breast cancer cell biology. a)** Circo plot displaying the genome distribution of the whole set of 5917 SEs that resulted from the analysis of H3K27ac density from ChIP-seq data from 17 non-transformed mammary epithelial and BCC lines. Each of the boxes in the inner circle represents an instance of a SE in one cell line. **b)** Number of SEs as a function of the number of cell lines where each SE is present. Line-specific SEs, present in only one cell line, are shown left to the red line. The inset shows a magnification of the last part of the plot (from 6 to 17 cell lines). Ubiquitous SEs are present in more than two thirds of the cell lines (12 or more), right to the red line. **c)** Representative significant GO Biological Process terms enriched for genes associated with ubiquitous SEs vs all SEs, analyzed by GREAT. The full list of terms is shown in **Suppl. Table 4. d)** Enrichment of ubiquitous SEs and depletion of line-specific SEs in the set of extended core regulatory circuitry (CRC) genes of three breast cell lines. The plot shows the odds-ratio with confidence intervals for a Fisher´s exact test (all *p*-values are < 0.05, except the one for MCF7 ubiquitous SEs). **e)** Analysis of differential activity for ubiquitous SE-associated master regulators (MR) of expression in breast cancer. Left: scheme of VIPER analysis. Subtypes: TNBC, Lum, HER2+ and normal (Nor). Right: regulators with a normalized enrichment score (NES) from VIPER analysis of more than 2 in absolute value, shown for each contrast (lines of the indicated subtype vs all the other lines). The color scale of the bars shows the change in expression of each MR for the indicated contrast. *ZBTB7B* (ThPOK) is indicated in red in the two contrasts where it appears (TNBC and Lum). **f)** Genomic context of *ZBTB7B* and its associated super-enhancer (red bar). The H3K27ac ChIP-seq signal is shown for all 17 breast cancer cell lines, grouped and colored by subtype.

**Figure 2. *ThPOK* expression is highest in breast cancer cell lines and tumors of the luminal subtype. a)** *ThPOK* expression in BCC lines measured by qRT-PCR. Bars and error bars represent the mean and the SD of ≥3 biological replicates. All values are referred to the average expression level in MCF10A cells. Breast cancer subtypes are colored as in Fig. 1: blue, Nor; orange, Lum; green, HER2+; brown, TNBC; red, *in vitro* transformed cells. **b)** *ThPOK* mean expression (in TPM) extracted from RNA-seq datasets listed in **Suppl. Table 5**. **c)** ThPOK expression assessed by immunofluorescence (red channel) in the indicated cell lines**.** Nuclei were counterstained with DAPI (blue channel). Images are shown at 400X magnification. **d) & e)** *ThPOK* expression in primary tumors (TCGA dataset). Statistical analysis was performed by using Student's *t*-Test with unequal variance. **d)** *ThPOK* expression is higher in primary tumors than in normal tissue, *: $p < 10^{-11}$. **e)** *ThPOK* expression in breast cancer subtypes. Luminal tumors express the highest *ThPOK* levels compared to normal tissue *: $p < 10^{-12}$, followed by HER2+ tumors, *: $p < 10^{-4}$. *ThPOK* expression in TNBC tumors is not significantly different to the levels in normal tissues. In **d & e** in parenthesis: number of tumors/normal tissues included in each subtype. These plots were generated using the UALCAN portal (http://ualcan.path.uab.edu). **f)** ThPOK expression is higher in cancer epithelial cells within tumor samples. *ThPOK* levels in 38241 cells from 11 ER+ tumor samples assessed by scRNA-seq. Cell types were assigned following the identities from the original paper.

**Figure 3. *ThPOK* knockdown dysregulates expression of ECM, EMT and WNT/β-catenin genes and increases migration in luminal BCCs. a, b, d-f, h-i)** Gene expression levels measured by qRT-PCR. Since three different shRNA against *ThPOK* were used, the circle for each gene is divided in thirds, one for each shRNA. The color of each third indicates the $\log_2$(fold-change) and the size the $-\log_{10}$(p-value), for a *t*-test relative to the control shRNA (shScr). Each gene was assessed in ≥3 biological replicates. Differential expression is shown in color scale as a log2 of the fold change, and *p*-values by the size of the circle as –log10. **c)** Bright field images show morphological changes associated with ThPOK-kd in Luminal and HER2+ cells. Scale: each side of the square corresponds to 400μm. **g)** Flow cytometry analysis showing CD44 expression in HER2+ scramble (Scr) and ThPOK-kd BT474 cells. **j-k)** Migration assays. **j)** Scratch assay showing increased migratory features of ThPOK-kd cells compared with control (scr) cells. Bar and error bars represent the mean and the SD of ≥3 biological replicates. *: $p < 10^{-6}$. **k)** Boyden chamber assay. Bar and errror bars represent the mean and the SD of ≥3 biological replicates, *: $p < 0.05$, **: $p < 10^{-4}$. All *p*-values in this figure were calculated based on a two-tailed Student's *t*-Test.

**Figure 4. *ThPOK* overexpression represses ECM, EMT and WNT/β-catenin genes and decreases migration in HER2+ and TNBC BCCs. a-g)** Gene expression levels measured by qRT-PCR. The $\log_2$(fold-change) in expression of each gene is shown in color scale and p-values by the size of the circle as $-\log_{10}$, relative to the empty vector control cells (EV). Each gene was assessed in ≥3 biological replicates. **h)** Bright field images show morphological changes associated with ThPOK overexpression in HER2+ and TNBC cells. Scale: each side of the square corresponds to 400μm. **g)** Boyden chamber migration assay. Absorbance of the crystal violet released by the migrating cells for each cell line is depicted as a percentage of the value corresponding to EV control cells. Bars and error bars represent the mean and SD of ≥3 biological replicates, *: $p < 10^{-6}$. *p*-value was calculated based on a two-tailed Student's *t*-Test.

**Figure 5. ThPOK represses TGFβ expression in breast cancer cell lines**. **a)** GSEA analysis shows an inverse correlation between ThPOK expression levels and TGFβ target genes. Genes were ordered according to their expression correlation with *ZBTB7B* using transcriptomic data from CCCL (breast cancer cell lines). TGFβ target genes are enriched at the end of the ordered set (negative correlations). **b) & c)** TGFβ (*TGFB1*) mRNA levels measured by qRT-PCR. **b)** TGFβ levels in ThPOK-kd luminal and HER2+ BCCs. *TGFB1* expression in each independent shRNA-containing cells is depicted as a triangle and the fold change is relative to that of the control shRNA (shScr). Each gene was assessed in ≥3 biological replicates. Differential expression is shown in color scale as a $\log_2$ of the fold change, and p-values by the size of the circle as $-\log_{10}$. **c)** TGFβ levels in HER2+ and TNBC cells overexpressing ThPOK ($ThPOK^{OE}$). *TGFB1* expression is shown as the fold change relative to that of the empty vector control cells (EV). Each gene was assessed in ≥3 biological replicates. Differential expression is shown in color scale as a $\log_2$ of the fold change, and p-values by the size of the circle as $-\log_{10}$. **d)** *FN1* relative mRNA levels measured by qRT-PCR in MCF7 cells after 3 days treatment with recombinant human TGFβ (rTGFβ), and T47D cells after 3 or 10 days treatment with rTGFβ. **e)** *FN1* relative mRNA levels measured by qRT-PCR in MDA-MB-231 and HCC1143 cells with $ThPOK^{OE}$ or EV, untreated (Untr) or treated with rTGFβ for 3 or 7 days. **f)** *FN1* and *COL1A1* relative mRNA levels measured by qRT-PCR in Scr and ThPOK-kd (kd) ZR75.1 and BT474 cells, treated with DMSO (Veh) or TGBRI inhibitor (LY). For **d**-**f**, all expression values are depicted relative to the mean of the corresponding controls. Bars and error bars represent the mean and the SD of ≥3 biological replicates. NS: non-significant; *: $p < 0.05$; **: $p < 0.005$; ***: $p < 0.0005$. All *p*-values in this figure were calculated based on a two-tailed Student's *t*-Test. **g)** Proposed model showing the direct and indirect regulation of extracellular matrix, EMT and TGFβ/SMAD target genes' expression by ThPOK. The size of the balloons represents the relative contribution (not in scale) of each factor to the cellular phenotypes.

## Figures

Figure 1

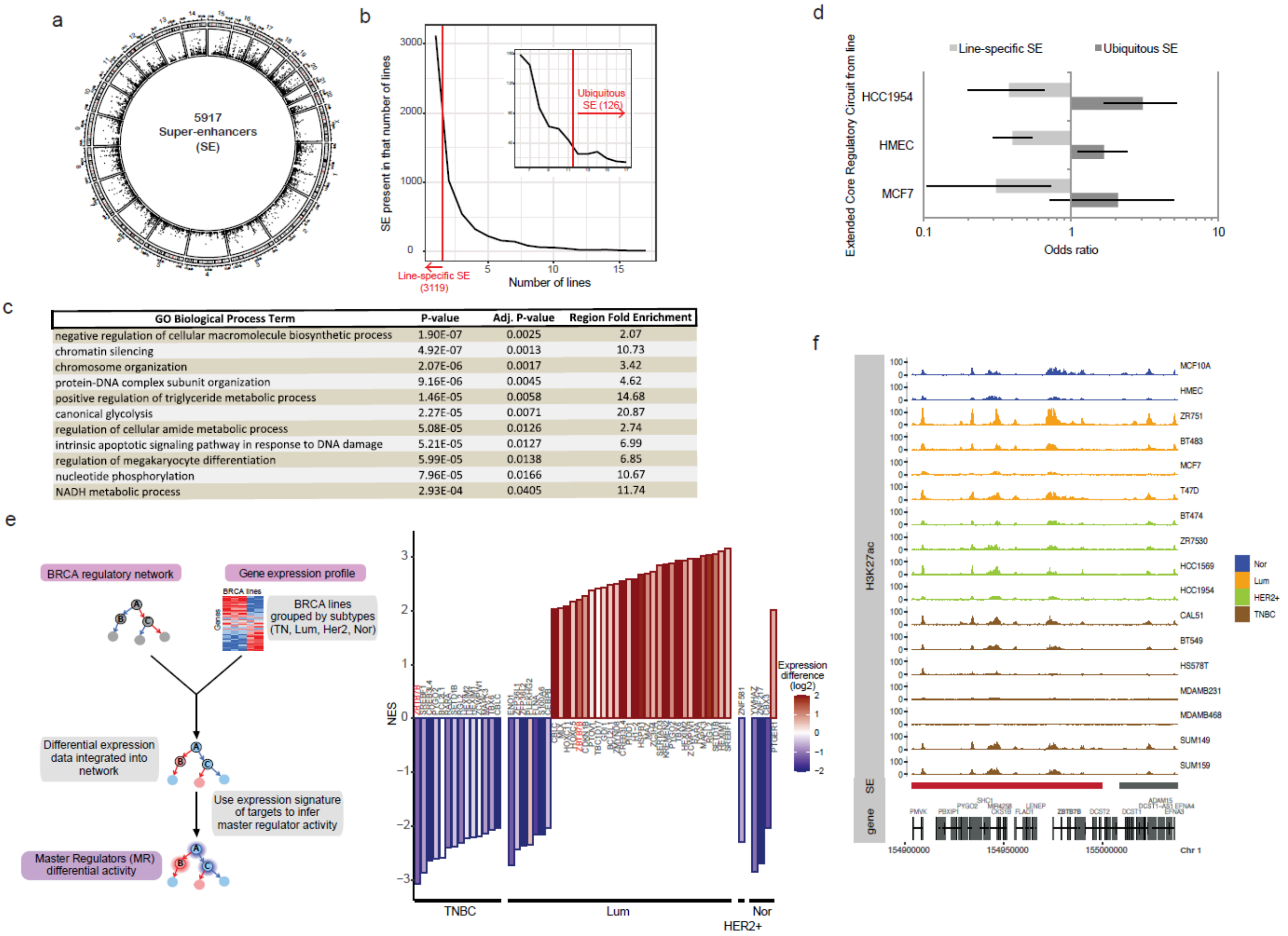

### Figure 1

Combining ubiquitous SE identification with subtype-specific master regulator activity analysis helps identification of candidate key regulators of breast cancer cell biology. a) Circo plot displaying the genome distribution of the whole set of 5917 SEs that resulted from the analysis of H3K27ac density from ChIP-seq data from 17 non-transformed mammary epithelial and BCC lines. Each of the boxes in the inner circle represents an instance of a SE in one cell line. b) Number of SEs as a function of the number of cell lines where each SE is present. Line-specific SEs, present in only one cell line, are shown left to the red line. The inset shows a magnification of the last part of the plot (from 6 to 17 cell lines). Ubiquitous SEs are present in more than two thirds of the cell lines (12 or more), right to the red line. c) Representative significant GO Biological Process terms enriched for genes associated with ubiquitous SEs vs all SEs, analyzed by GREAT. The full list of terms is shown in Suppl. Table 4. d) Enrichment of ubiquitous SEs and depletion of line-specific SEs in the set of extended core regulatory circuitry (CRC) genes of three breast cell lines. The plot shows the odds-ratio with confidence intervals for a Fisher´s exact test (all p-values are < 0.05, except the one for MCF7 ubiquitous SEs). e) Analysis of differential activity for ubiquitous SE-associated master regulators (MR) of expression in breast cancer. Left: scheme of VIPER analysis. Subtypes: TNBC, Lum, HER2+ and normal (Nor). Right: regulators with a normalized enrichment score (NES) from VIPER analysis of more than 2 in absolute value, shown for each contrast (lines of the indicated subtype vs all the other lines). The color scale of the bars shows the change in expression of each MR for the indicated contrast. ZBTB7B (ThPOK) is indicated in red in the two contrasts where it appears (TNBC and Lum). f) Genomic context of ZBTB7B and its associated super-enhancer (red bar). The H3K27ac ChIP-seq signal is shown for all 17 breast cancer cell lines, grouped and colored by subtype.

Figure 2

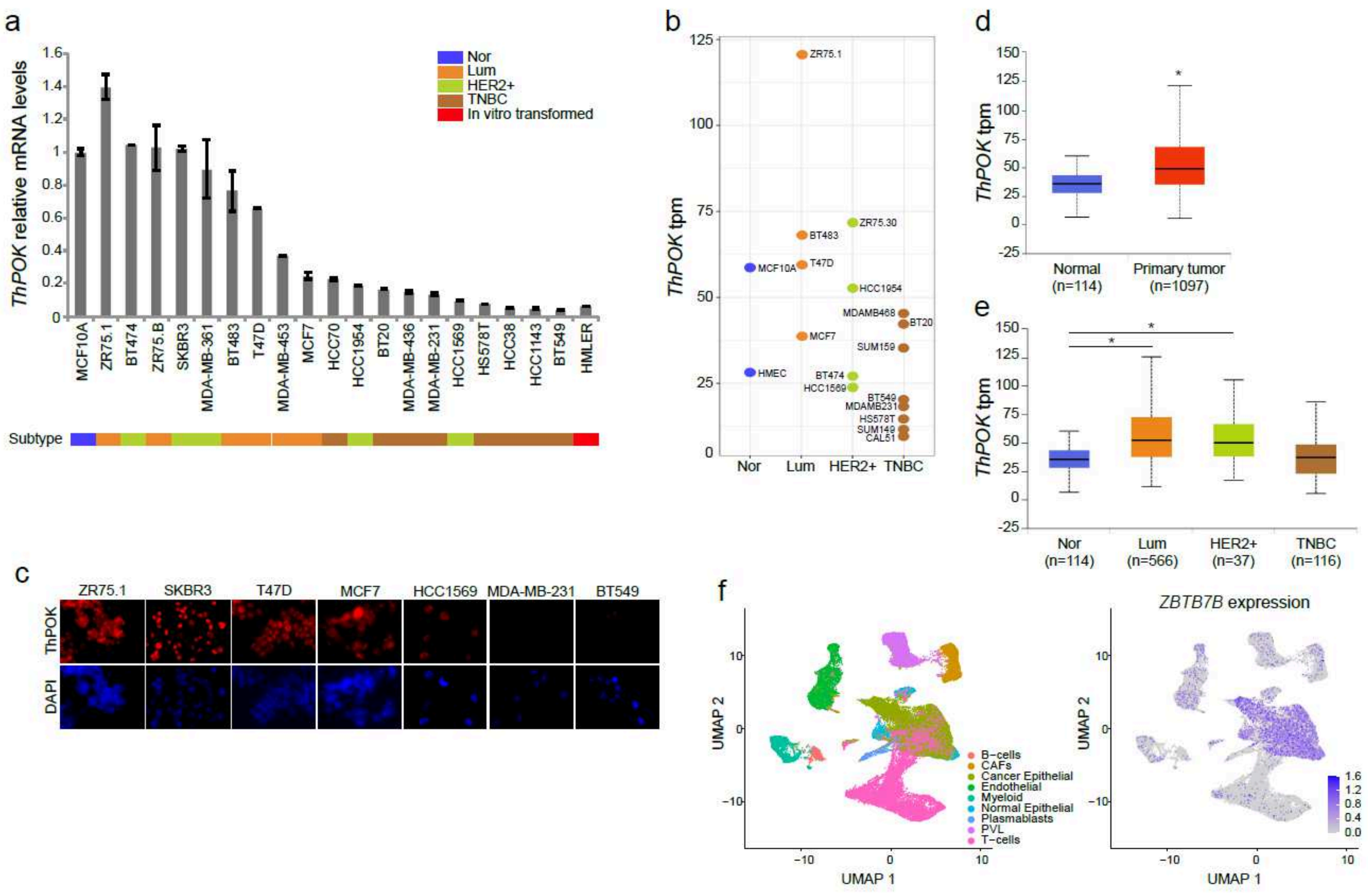

### Figure 2

ThPOK expression is highest in breast cancer cell lines and tumors of the luminal subtype. a) ThPOK expression in BCC lines measured by qRT-PCR. Bars and error bars represent the mean and the SD of ≥3 biological replicates. All values are referred to the average expression level in MCF10A cells. Breast cancer subtypes are colored as in Fig. 1: blue, Nor; orange, Lum; green, HER2+; brown, TNBC; red, in vitro transformed cells. b) ThPOK mean expression (in TPM) extracted from RNA-seq datasets listed in Suppl. Table 5. c) ThPOK expression assessed by immunofluorescence (red channel) in the indicated cell lines. Nuclei were counterstained with DAPI (blue channel). Images are shown at 400X magnification. d) & e)

ThPOK expression in primary tumors (TCGA dataset). Statistical analysis was performed by using Student's t-Test with unequal variance. d) ThPOK expression is higher in primary tumors than in normal tissue, *: $p < 10-11$. e) ThPOK expression in breast cancer subtypes. Luminal tumors express the highest ThPOK levels compared to normal tissue *: $p < 10-12$, followed by HER2+ tumors, *: $p < 10-4$. ThPOK expression in TNBC tumors is not significantly different to the levels in normal tissues. In d & e in parenthesis: number of tumors/normal tissues included in each subtype. These plots were generated using the UALCAN portal (http://ualcan.path.uab.edu). f) ThPOK expression is higher in cancer epithelial cells within tumor samples. ThPOK levels in 38241 cells from 11 ER+ tumor samples assessed by scRNA-seq. Cell types were assigned following the identities from the original paper.

Figure 3

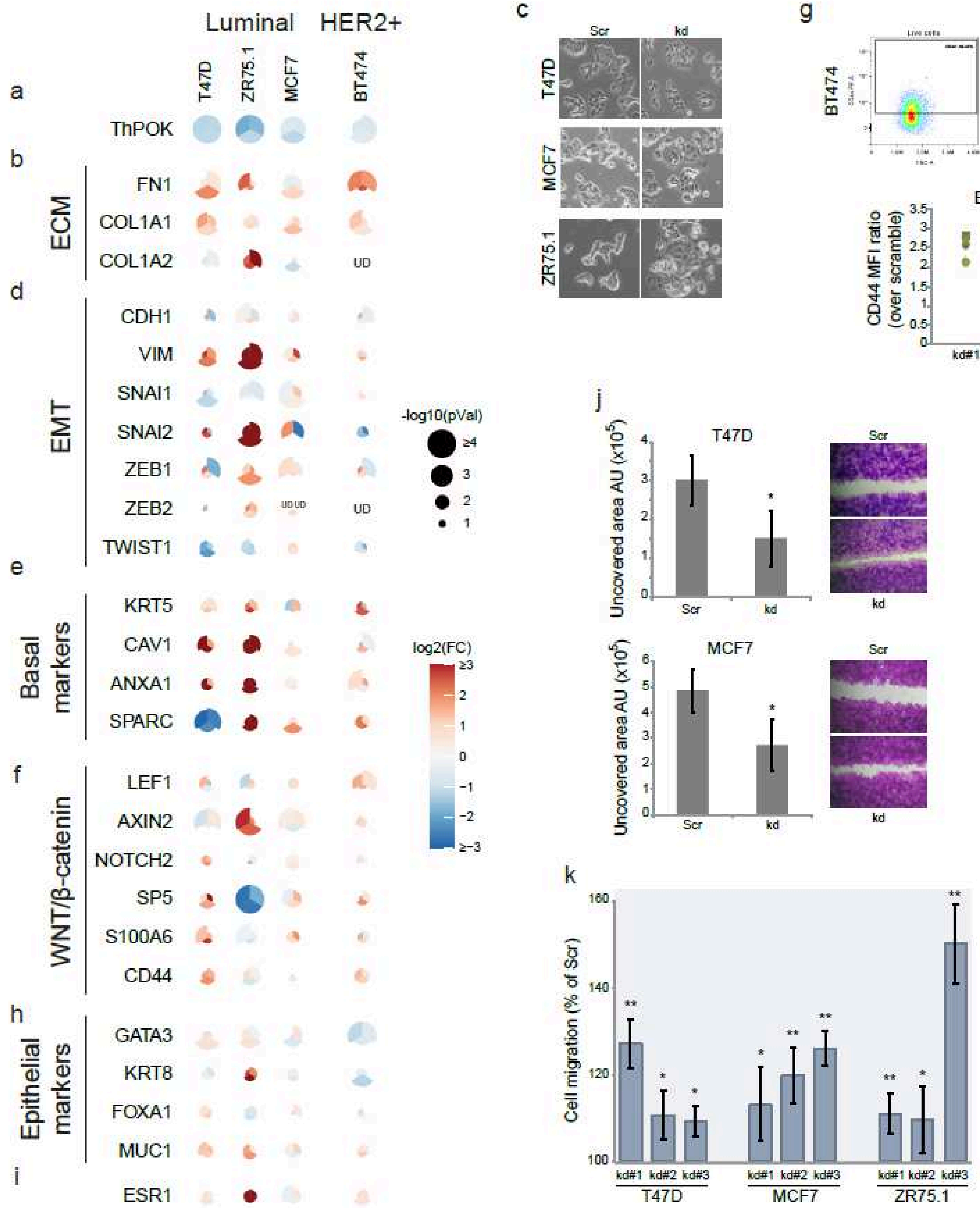

### Figure 3

ThPOK knockdown dysregulates expression of ECM, EMT and WNT/bcatenin genes and increases migration in luminal BCCs. a, b, d-f, h-i) Gene expression levels measured by qRT-PCR. Since three different shRNA against ThPOK were used, the circle for each gene is divided in thirds, one for each shRNA. The color of each third indicates the log2(fold-change) and the size the -log10(p-value), for a t-test relative to the control shRNA (shScr). Each gene was assessed in ≥3 biological replicates.

Differential expression is shown in color scale as a log2 of the fold change, and p-values by the size of the circle as –log10. c) Bright field images show morphological changes associated with ThPOK-kd in Luminal and HER2+ cells. Scale: each side of the square corresponds to 400μm. g) Flow cytometry analysis showing CD44 expression in HER2+ scramble (Scr) and ThPOK-kd BT474 cells. j-k) Migration assays. j) Scratch assay showing increased migratory features of ThPOK-kd cells compared with control (scr) cells. Bar and error bars represent the mean and the SD of ≥3 biological replicates. *: $p < 10-6$. k) Boyden chamber assay. Bar and errror bars represent the mean and the SD of ≥3 biological replicates, *: $p < 0.05$, **: $p < 10-4$. All pvalues in this figure were calculated based on a two-tailed Student’s t-Test.

Figure 4

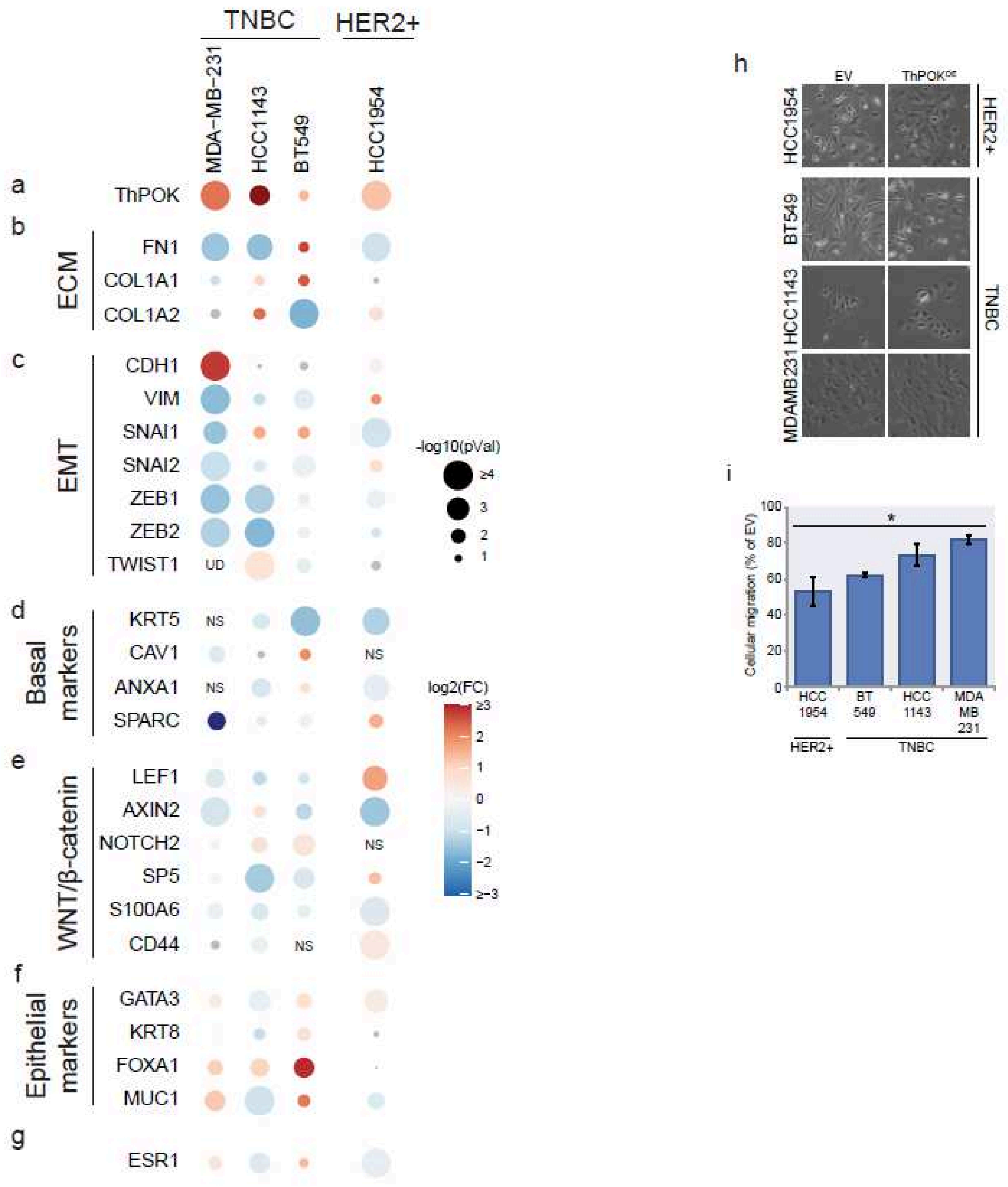

### Figure 4

ThPOK overexpression represses ECM, EMT and WNT/b-catenin genes and decreases migration in HER2+ and TNBC BCCs. a-g) Gene expression levels measured by qRT-PCR. The log2(fold-change) in expression of each gene is shown in color scale and p-values by the size of the circle as –log10, relative to the empty vector control cells (EV). Each gene was assessed in ≥3 biological replicates. h) Bright field images show morphological changes associated with ThPOK overexpression in HER2+ and TNBC cells.

Scale: each side of the square corresponds to 400µm. g) Boyden chamber migration assay. Absorbance of the crystal violet released by the migrating cells for each cell line is depicted as a percentage of the value corresponding to EV control cells. Bars and error bars represent the mean and SD of ≥3 biological replicates, *: p < 10-6. p-value was calculated based on a two-tailed Student’s t-Test.

Figure 5

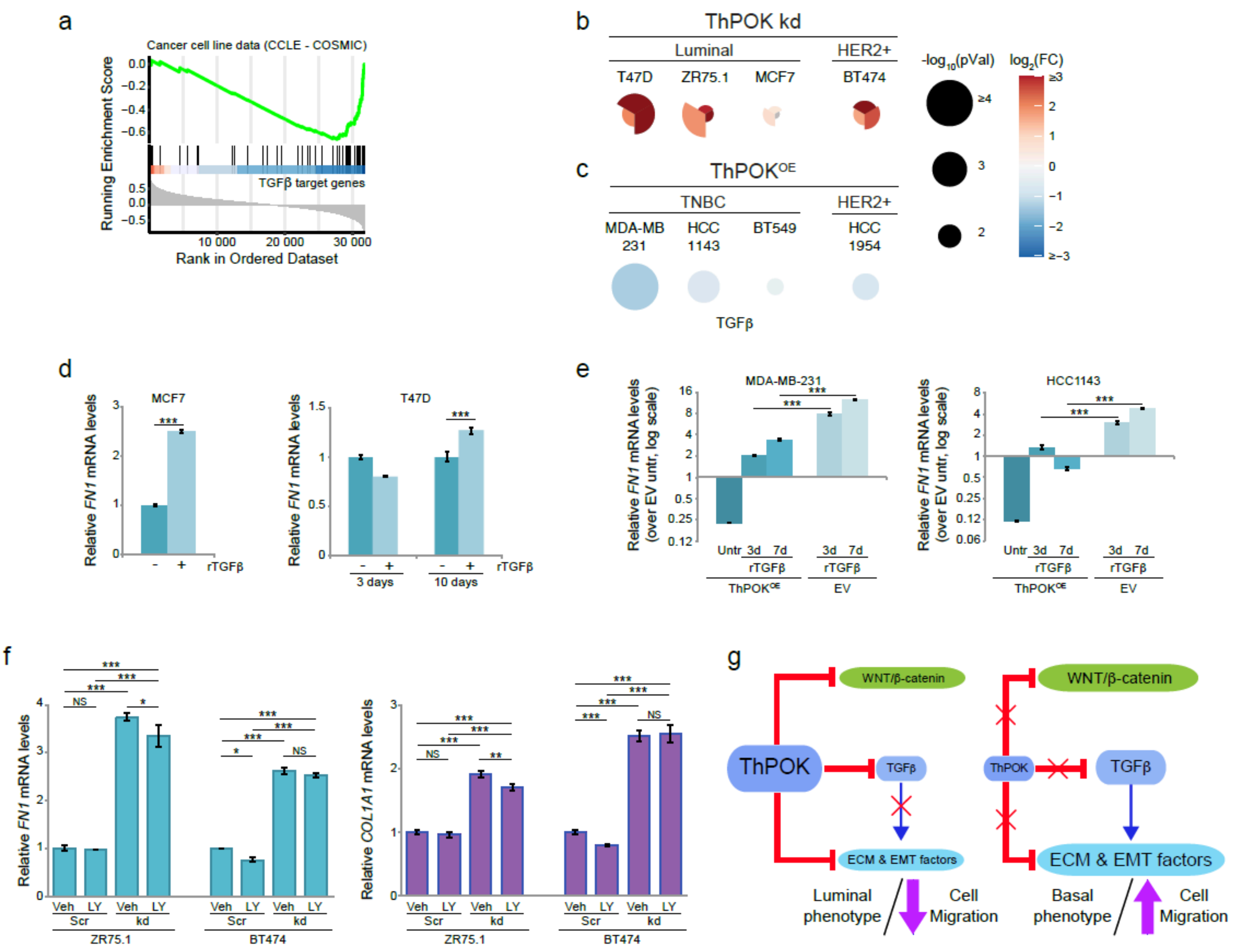

**Figure 5**

ThPOK represses TGFb expression in breast cancer cell lines. a) GSEA analysis shows an inverse correlation between ThPOK expression levels and TGFb target genes. Genes were ordered according to their expression correlation with ZBTB7B using transcriptomic data from CCCL (breast cancer cell lines). TGFb target genes are enriched at the end of the ordered set (negative correlations). b) & c) TGFb (TGFB1) mRNA levels measured by qRT-PCR. b) TGFb levels in ThPOK-kd luminal and HER2+ BCCs. TGFB1 expression in each independent shRNA-containing cells is depicted as a triangle and the fold change is relative to that of the control shRNA (shScr). Each gene was assessed in ≥3 biological replicates. Differential expression is shown in color scale as a log2 of the fold change, and p-values by the size of the circle as –log10. c) TGFb levels in HER2+ and TNBC cells overexpressing ThPOK (ThPOKOE). TGFB1 expression is shown as the fold change relative to that of the empty vector control cells (EV). Each gene was assessed in ≥3 biological replicates. Differential expression is shown in color scale as a log2 of the fold change, and p-values by the size of the circle as –log10. d) FN1 relative mRNA levels measured by qRT-PCR in MCF7 cells after 3 days treatment with recombinant human TGFb (rTGFb), and T47D cells after 3 or 10 days treatment with rTGFb. e) FN1 relative mRNA levels measured by qRTPCR in MDA-MB-231 and HCC1143 cells with ThPOKOE or EV, untreated (Untr) or treated with rTGFb for 3 or 7 days. f) FN1 and COL1A1 relative mRNA levels measured by qRT-PCR in Scr and ThPOK-kd (kd) ZR75.1 and BT474 cells, treated with DMSO (Veh) or TGBRI inhibitor (LY). For d-f, all expression values are depicted relative to the mean of the corresponding controls. Bars and error bars represent the mean and the SD of ≥3 biological replicates. NS: non-significant; *: $p < 0.05$; **: $p < 0.005$; ***: $p < 0.0005$. All p-values in this figure were calculated based on a two-tailed Student's t-Test. g) Proposed model showing the direct and indirect regulation of extracellular matrix, EMT and TGFb/SMAD target genes' expression by ThPOK. The size of the balloons represents the relative contribution (not in scale) of each factor to the cellular phenotypes.

## Supplementary Files

This is a list of supplementary files associated with this preprint. Click to download.

- SupplementaryFig.1.pdf
- SupplementaryFig.2.pdf
- SupplementaryFig.3.pdf
- SupplementaryFig.4.pdf
- SupplementaryFig.5.pdf
- SupplementaryFig.6.pdf
- SupplementaryFig.7.pdf
- SupplementaryFig.8.pdf
- SupplementaryFig.9.pdf
- SupplementaryTable1.xlsx
- SupplementaryTable2.xlsx
- SupplementaryTable3.xlsx
- SupplementaryTable4.xlsx
- SupplementaryTable5.xlsx
- SupplementaryTable6.xlsx
- SupplementarymaterialThPOKpaperfinal.pdf
